# Vacuum-Ultraviolet-Induced Fragmentation of Furan Studied by Photon-Induced Fluorescence Spectroscopy

**DOI:** 10.3390/molecules31213898

**Published:** 2026-10-29

**Authors:** Tomasz J. Wasowicz, Marcin Dampc, Marcello Coreno, Antti Kivimäki

**Affiliations:** 1 Division of Complex Systems Spectroscopy, Institute of Physics and Applied Computer Science, Faculty of Applied Physics and Mathematics, Gdansk University of Technology, ul. G. Narutowicza 11/12, 80-233 Gdansk, Poland; 2 CNR—Istituto di Struttura della Materia (ISM), in Basovizza Area Science Park, 34149 Trieste, Italy; 3 MAX IV Laboratory, Lund University, P.O. Box 118, 221 00 Lund, Sweden

**Keywords:** furan, vacuum ultraviolet, VUV photodissociation, photon-induced fluorescence spectroscopy, synchrotron radiation, UV-visible emission, electronically excited neutral fragments, superexcited states, radiation chemistry, biofuel chemistry

## Abstract

Photodissociation of furan was investigated by photon-induced fluorescence spectroscopy using synchrotron radiation over the photon-energy range of 17–47 eV. Dispersed emission spectra recorded at a higher energy, 62 eV, revealed electronically excited hydrogen atoms, CH(*A*^2^Δ, *B*^2^Σ^−^, *C*^2^Σ^+^) radicals, and C_2_(*d*^3^Π_g_) fragments as the dominant emissive photodissociation products observed under the present experimental conditions. Emission yield dependences of the H(*n* = 3–6), CH(*A*^2^Δ), and C_2_(*d*^3^Π_g_) channels were measured and parameterized using a phenomenological multi-Gaussian decomposition to characterize broad photon-energy regions contributing to the observed emissions. Appearance energies of the H(*n* = 3–6) and C_2_(*d*^3^Π_g_) channels were determined from threshold analyses and compared with estimated dissociation energy limits. The observed threshold shifts and photon energy dependences indicate that the formation of the emitting fragments involves multistep dissociation pathways, likely including ring opening and hydrogen migration processes. Relative fragment emission yields were further examined to probe competition between different emissive dissociation channels. Hydrogen, CH, and C_2_ emissions exhibit distinct photon energy dependences, indicating that different regions of the excited-state manifold preferentially populate different fragmentation pathways. The present work provides new insight into the formation of electronically excited neutral fragments following vacuum-ultraviolet (VUV) excitation of furan and demonstrates that fragment selective optical emission spectroscopy offers a useful approach for investigating the dissociation of highly excited molecular states in heterocyclic molecules.

## 1. Introduction

**Figure 1:**
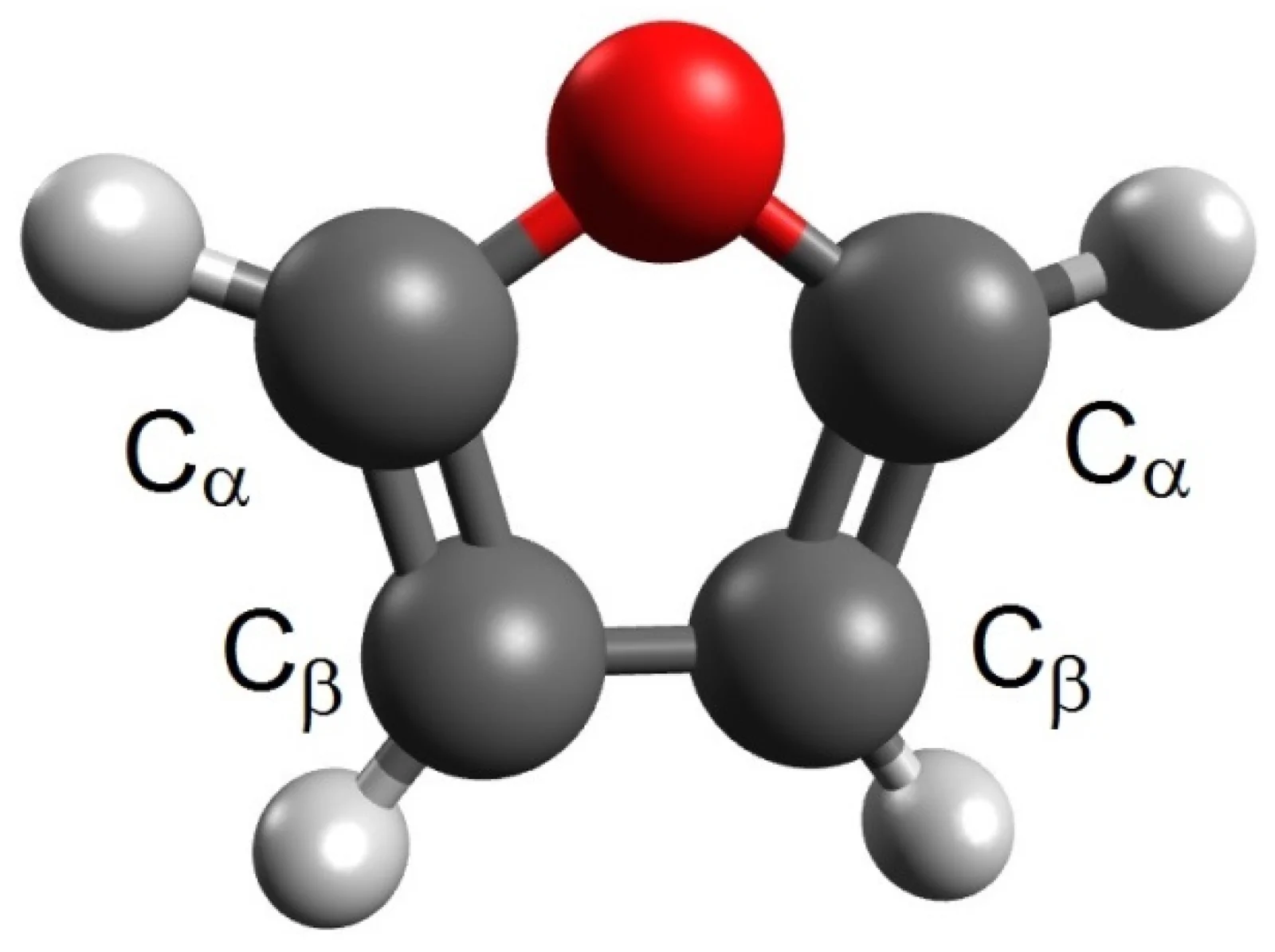
Molecular structure of furan (C_4_H_4_O) [8]. Carbon atoms are shown in dark grey, oxygen in dark red, and hydrogen in light grey.

Furan (C_4_H_4_O) is one of the most fundamental five-membered heterocyclic aromatic compounds and represents a basic structural motif in a wide range of organic and biologically active substances. Its five-membered ring, composed of four carbon atoms and one oxygen atom (see Figure 1), is regarded as a building unit in vitamin B_12_ [1], organic synthesis products [2], and in several classes of conducting polymers [3]. The five-membered oxygen-containing ring of furan provides a structural analogy to the furanose forms of ribose and deoxyribose in RNA and DNA [4]. Furan-substituted compounds are used as antibiotics [5] and in the synthesis of prodrugs employed in cancer therapy [6]. Therefore, studies of furan fragmentation provide a model for examining radiation-induced processes in oxygen-containing heterocyclic molecules [7].

In addition to its biological relevance, furan and its derivatives are widely used in industrial and technological processes. Furan derivatives are also used in photopolymerizable resins for stereolithography [9] and investigated as biomass-derived fuel components [10,11]. Furan has also been detected in heat-treated foods as a product of the thermal degradation of natural food constituents [12,13].

Furan has a relatively low first ionization energy of about 8.9 eV, as determined by threshold and Rydberg-series photoelectron spectroscopy [14,15]. Owing to the presence of well-characterized valence and Rydberg states, furan has become a benchmark system for the investigation of electronic excitation and relaxation processes in heteroaromatic molecules [16]. Time-resolved and spectroscopic studies in the vacuum-ultraviolet and ultraviolet regions have demonstrated that furan exhibits absorption bands associated with valence and Rydberg excitations and undergoes ultrafast non-adiabatic relaxation following photoexcitation [17,18].

Early studies of furan fragmentation focused on the kinetics and mechanisms of thermal unimolecular decomposition. Grela et al. [19] employed a very-low-pressure flow reactor to examine furan pyrolysis. Complementary studies were performed by Lifshitz et al. [20] and by Organ et al. [21] using single-pulse shock-tube techniques in the temperature ranges of 1050–1460 K and 1100–1700 K, respectively. Later, these investigations were extended over a broad temperature interval (500–3000 K) by combining shock-wave excitation with laser-schlieren densitometry and time-of-flight mass spectrometry [22]. The reported product channels included the formation of CO together with propyne (CH_3_C≡CH), acetylene (HC≡CH) together with ketene (H_2_C=C=O), and the radical pair HCO and propargyl (HC≡C–CH_2_). Organ et al. [21] further proposed that the dissociation is initiated by intramolecular hydrogen migration followed by cleavage of the O–C_α_ bond, leading to a linear carbene (L-furan) intermediate that subsequently decomposes into the observed fragments. This mechanism was later supported by ab initio calculations of unimolecular decomposition pathways [23]. Vasiliou et al. [24] detected aromatic products such as benzene and styrene during furan pyrolysis using photoionization mass spectrometry and matrix-isolation infrared spectroscopy.

Photoionization and ion-fragment studies have provided extensive information on furan fragmentation [25,26,27], but less is known about the formation of electronically excited neutral fragments via photoabsorption and the excitation mechanisms involved. Optical emission spectroscopy provides complementary information by detecting the excited dissociation products. In optical-emission studies of electron-induced fragmentation, Dampc and Zubek [28] observed excited hydrogen atoms H(*n*) as well as vibrationally and rotationally excited CH(*A*^2^Δ), CH(*B*^2^Σ^−^), CH(*C*^2^Σ^+^), and C_2_(*d*^3^Π_g_) fragments. They suggested that fragmentation proceeds through opening of the furan ring to form an excited oxy-1,3-butadienylene diradical intermediate, •CH=CH–CH=CH–O•, which subsequently dissociates. The proposed fragmentation channels leave approximately 2–5 eV available for translational and internal excitation of the fragments. Dampc et al. [29] measured electron-impact ionization and cation fragmentation of gas-phase furan, identifying the parent C_4_H_4_O^+^ and primary C_3_H_3_^+^ fragments, and providing absolute total and partial ionization cross sections up to 150 eV. Studies of dissociative electron attachment to furan and related molecules have also revealed efficient low-energy electron-induced bond cleavage [30]. Moreover, the C–H bond dissociation energy in furan has been established using photoelectron spectroscopy of the furanide anion [31].

Emission from electronically excited neutral fragments of furan has also been investigated in ion–molecule collisions with He^+^, He^2+^, H^+^, and H_2_^+^ projectiles [32,33,34,35]. These studies identified several emitting fragments, including OH, whose formation provided evidence of intramolecular hydrogen migration [35]. Further experiments with H_3_^+^ and C^+^ projectiles revealed charge-transfer and reactive fragmentation channels [36]. Complementary studies by Erdmann et al. investigated furan reactions with H_2_O^+^ and OH^+^ ions, reporting absolute reaction cross sections and product distributions relevant to radiation chemistry [37].

Sendt et al. [38] performed ab initio calculations of the potential-energy surface of furan and its isomers and decomposition pathways. Their results suggested that simple direct cleavage of the C–O or C–H bonds is unlikely to represent the primary dissociation step. Instead, they proposed that hydrogen migration within cyclic carbene intermediates constitutes the first step of the dissociation process. A closely related mechanism was obtained in ab initio and semi-empirical modelling of furan photoion fragmentation following valence-shell ionization with 11.6–13.5 eV photons [39]. Those calculations demonstrated that the initial step of parent-ion dissociation is an isomerization involving hydrogen transfer from C_α_ to C_β_, which is energetically more favorable than direct bond fission. Erdmann et al. [40] studied the unimolecular decomposition of furan using high-level electronic-structure methods and statistical rate modelling. They identified the energetically most favorable reaction pathways and quantified the corresponding activation barriers for ring opening, isomerization and subsequent fragmentation steps. Their results showed competition between rearrangement and direct bond fission over a wide internal-energy range. Further theoretical analysis [41] provided a reaction network for furan pyrolysis and demonstrated that isomerization and ring-opening reactions leading to open-chain intermediates play an important role in initiating decomposition. The calculated rate constants and dominant reaction pathways were in good agreement with available shock-tube and pyrolysis experiments, further supporting the view that rearrangement-driven mechanisms govern the primary steps of furan fragmentation. The calculated pathways leading to OH formation [40,41] were additionally supported by the observation of OH emission in ion–molecule collisions [35].

At photon energies above the first ionization threshold, furan can be excited into neutral superexcited states [42]. These states may have highly excited Rydberg, doubly excited valence, or inner-valence character and can undergo autoionization or dissociation [42,43]. Dissociation of such states can lead to the formation of electronically excited neutral fragments. However, the dissociation of highly excited states of furan into specific atomic and molecular fragments remains largely unexplored. In the present work, dispersed photon-induced emission spectroscopy is used to probe electronically excited fragments formed during VUV-induced dissociation of furan. The combined analysis of the emission yields and dispersed spectra provides insight into the pathways leading to the formation of these fragments following VUV excitation of furan. The results indicate that molecular rearrangement plays an important role in the formation of the observed excited fragments, although the extent of structural reorganization appears to differ among the various dissociation channels.

## 2. Results and Discussion

### 2.1. Optical Emission Spectrum

**Figure 2:**
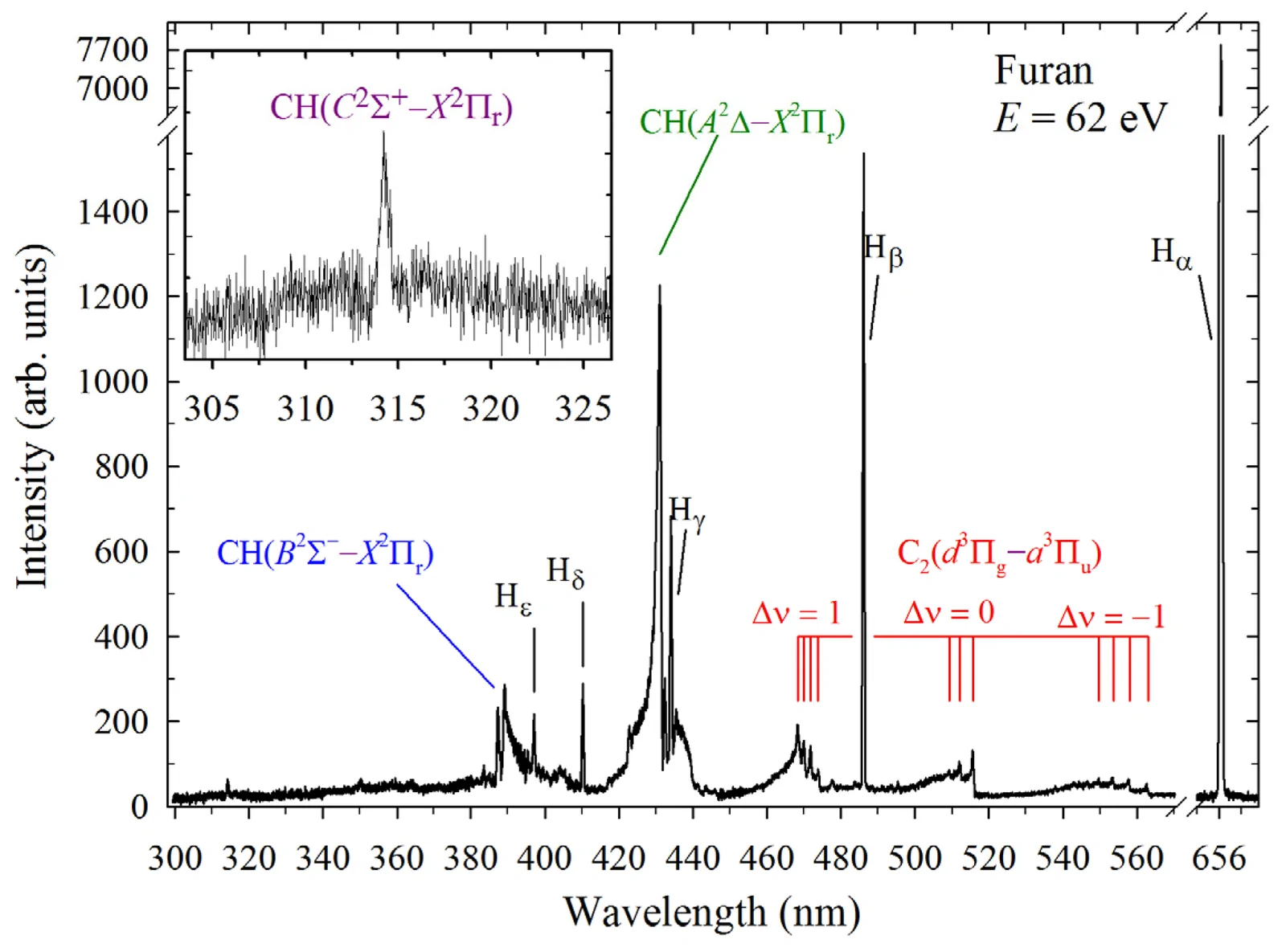
Dispersed fluorescence spectrum of furan recorded at photon energy of 62 eV in the wavelength range 300–660 nm. The observed emissions include atomic hydrogen Balmer lines together with molecular-fragment bands of CH and C_2_. The inset shows an expanded view of the 304–326 nm spectral region containing the weak CH(*C*^2^Σ^+^) emission bands. The spectrum is presented primarily for identification of the emitting fragments observed in the experiment.

Figure 2 shows the optical emission spectrum recorded for the photodissociation of furan at the photon energy of 62 eV in the 300–660 nm wavelength range. The 62 eV spectrum is used here primarily to identify the emissive fragments and is not used to infer the dominant excitation mechanism in the 17–48 eV excitation-function measurements. The CH(*A*^2^Δ → *X*^2^Π_r_) system near 431 nm is the strongest molecular emission observed in the spectrum. This band originates from radiative relaxation of electronically excited CH radicals formed during fragmentation of the parent molecule. Its broad and asymmetric profile results from overlapping rotational and vibrational branches of the A–X transition. A weaker CH(*B*^2^Σ^−^ → *X*^2^Π_r_) emission band is observed, with the most pronounced structure near 387 nm. The presence of the B–X system confirms population of higher electronically excited states of the CH fragment during the dissociation process. Compared with the intense CH(A–X) emission, the B–X transition exhibits significantly lower intensity, indicating less efficient production of CH radicals in the *B*^2^Σ^−^ state. In the ultraviolet range between approximately 305 and 325 nm, the spectrum also reveals the CH(*C*^2^Σ^+^ → X^2^Π_r_) system shown in the inset. The emission is associated mainly with transitions belonging to the Δν = 0 sequence. The strongest contribution corresponds to the (0, 0) vibrational band near 314.5 nm, while weaker asymmetric structures around 312 and 316 nm arise from overlapping rotational branches of neighboring vibrational transitions. Several Balmer lines due to excited atomic hydrogen are present in the visible region, indicating dissociation pathways involving C–H bond cleavage and formation of excited hydrogen atoms. The strongest hydrogen feature corresponds to Hα at 656.3 nm. Additional weaker lines identified as Hβ (486.1 nm), Hγ (434.0 nm), Hδ (410.2 nm), and Hε (397.0 nm) are also visible in the spectrum. Their relative intensities decrease systematically with increasing principal quantum number *n*. Weak molecular structures observed between about 450 and 570 nm are assigned to the C_2_ (*d*^3^Π_g_ → *a*^3^Π_u_) Swan system. Bands belonging to the Δν = 0, +1 and −1 vibrational sequences can be distinguished.

Dispersed fluorescence spectrum of furan measured at 62 eV exhibits several features previously reported in electron- and ion-induced fragmentation studies of this molecule [28,32,33,34,35,36]. The coexistence of these emissions indicates that electronically excited CH radicals and hydrogen atoms constitute the dominant neutral fragments formed during furan decomposition. The present spectrum shows neither the weak atomic carbon and helium lines observed in some collisional systems nor the OH(*A*^2^Σ^+^ → *X*^2^Π) emission attributed to hydrogen migration processes [32,33,34,35,36].

These spectral differences may result from different primary excitation mechanism and the subsequent fragmentation dynamics. At a photon energy of 62 eV, dissociative decay following valence-shell photoionization is expected to make a predominant contribution to the observed fluorescence, as discussed in Section 2.5.

### 2.2. Emission Yields

**Figure 3:**
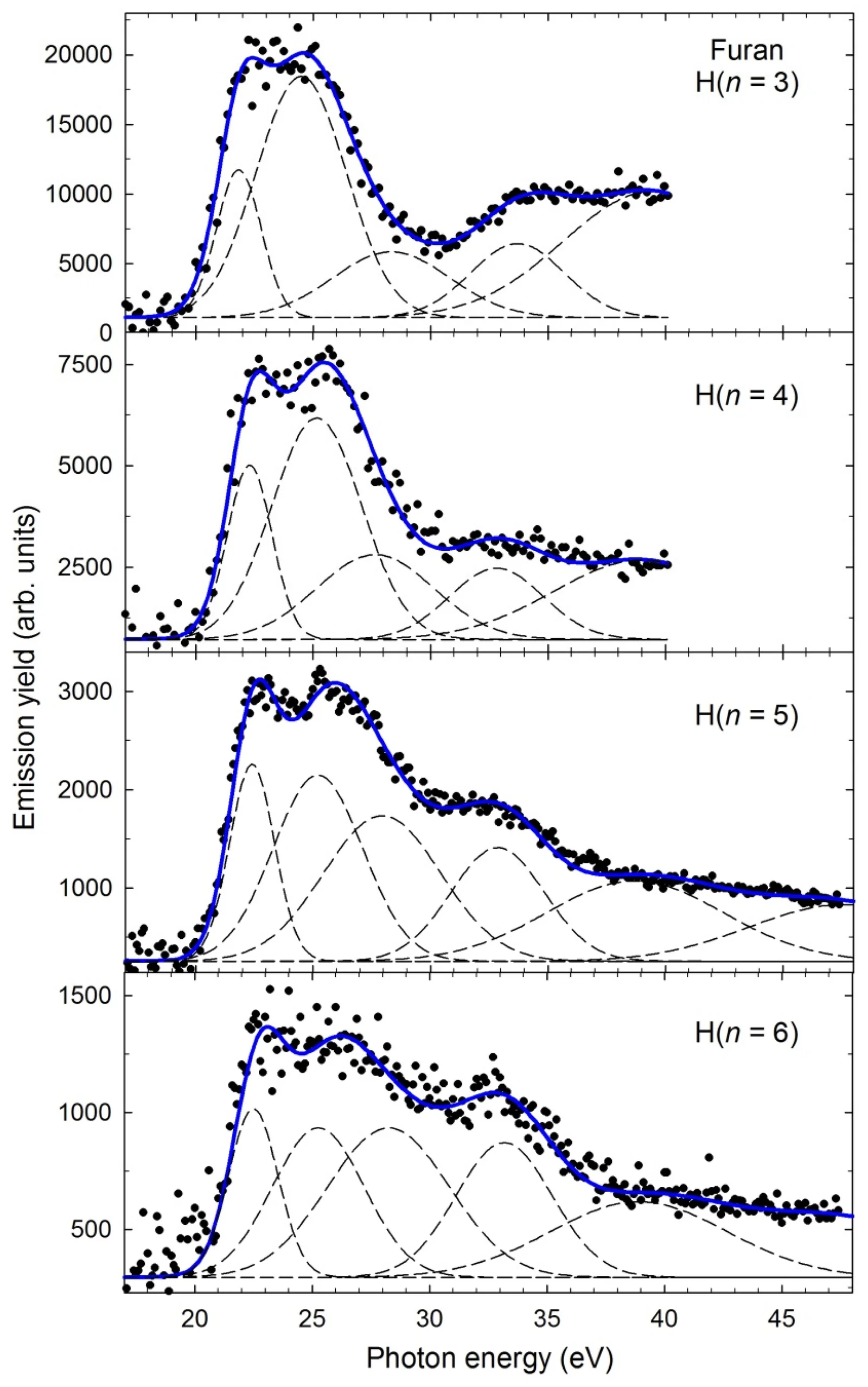
Emission yield curves of the Balmer-series emissions H(*n* = 3–6) produced in the VUV-induced dissociation of furan are shown together with the corresponding multi-component Gaussian fits. Solid dots represent the experimental data, while solid lines show the multi-component Gaussian fits. Dashed lines represent the individual Gaussian components.

**Figure 4:**
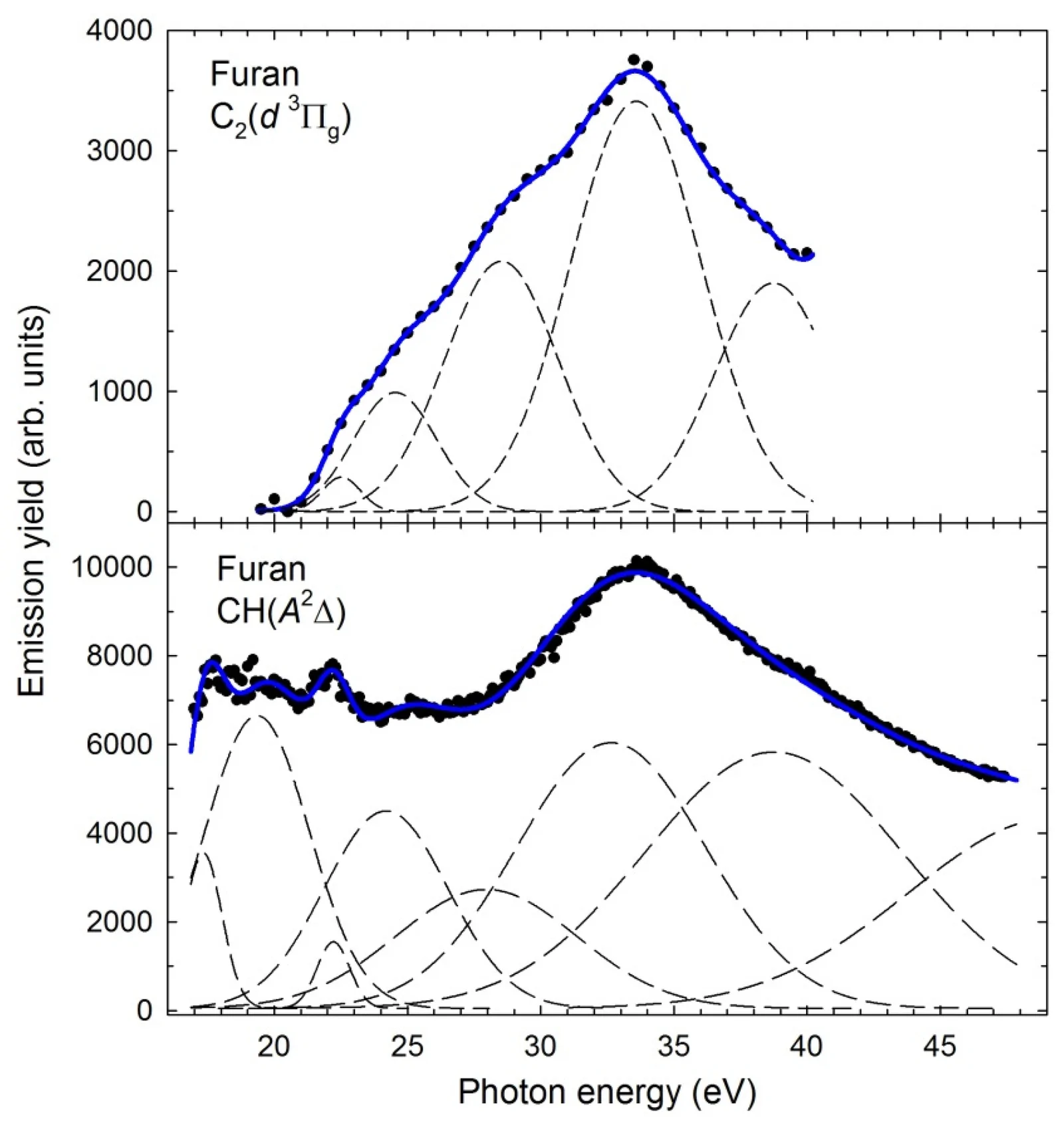
Emission yield curves of the C_2_(*d*^3^Π_g_) and CH(*A*^2^Δ) emissions produced in the VUV-induced dissociation of furan are shown together with the corresponding multi-component Gaussian fits. Solid dots represent the experimental data, while solid lines show the multi-component Gaussian fits. Dashed lines represent the individual Gaussian components.

The photon energy dependences of the emission yield of selected transitions are shown in Figure 3 and Figure 4. To analyze them, the experimental curves were decomposed into a set of Gaussian components using a least-squares fitting procedure. Initial values of the band positions and widths were estimated from the dominant structures observed in the excitation functions. The fits were performed over partially overlapping energy intervals in order to reduce the number of simultaneously optimized parameters. For corresponding structures observed in some different fragment channels, common bandwidths were used during the fitting procedure.

The resulting Gaussian components should not be interpreted as distinct electronic states, but rather as broad excitation energy regions associated with overlapping excited-state manifolds contributing to the observed fragmentation dynamics. The relative intensities and energy distributions of the Gaussian components are therefore treated as phenomenological descriptors of fragment production pathways rather than state-specific branching ratios. The characteristic energy regions obtained from the Gaussian decomposition of the emission yield curves for the H(*n*), CH(*A*^2^Δ), and C_2_(*d*^3^Π_g_) fragmentation channels are summarized in Table 1 and Table 2. The quoted energies correspond to the maxima of the fitted Gaussian components, while the uncertainties were estimated from the fitting procedure. For the H(*n* = 3–6) emission channels, the characteristic energies of 28.0 and 33.1 eV represent the mean positions of the corresponding Gaussian components fitted to the four Balmer emission-yield curves. These values may differ from the maxima of the total emission-yield curves because the individual Gaussian components overlap.

The emission yield curves of the Balmer-series emissions H(*n* = 3–6), Hα-Hδ, as seen in Figure 3, show a similar overall excitation-energy dependence, with a pronounced increase in intensity above approximately 20 eV and broad structures extending over the 20–40 eV region. The Hα emission is the dominant channel throughout the investigated energy range, while the relative contributions of the Balmer lines with *n* ≥ 4 vary significantly with excitation energy. In particular, the Hγ and Hδ channels become relatively more intense in the 22–32 eV region, indicating changes in the dissociation dynamics and in the distribution of excitation energy among the hydrogen fragments. Above approximately 33 eV, the emission yield profiles become smoother and less structured, which may reflect increasing contributions from overlapping excited ionic states. As discussed in Section 2.5, the broad energy distribution of correlation satellite states accompanying valence photoionization may contribute to such smooth excitation profiles.

Figure 4 presents the photon energy dependences of the CH(*A*^2^Δ), and C_2_(*d*^3^Π_g_) emission yields measured for the VUV-induced dissociation of furan together with the corresponding multi component Gaussian fits. In contrast to the Balmer series emissions shown in Figure 3, the molecular fragment emission profiles exhibit broader and less regular structures extending over a wider excitation energy range. The individual Gaussian components listed in Table 2 represent overlapping excitation energy regions associated with different excited state manifolds contributing to the formation of the observed molecular fragments.

The CH(*A*^2^Δ) emission is characterized by a broad and complex excitation profile extending throughout the entire energy range investigated. Based on previous PIFS studies of tetrahydrofuran [45] and isoxazole [46], the appearance threshold for CH(A–X) formation is expected to lie around 14–15 eV, i.e., below the lower limit of the present measurements (17 eV). Consequently, the CH(A–X) emission is already well established at the lowest photon energies covered in the present experiment. Several overlapping structures are visible between approximately 18 and 40 eV, indicating contributions from multiple dissociation pathways and extensive overlap of the excited-state manifolds involved in CH fragment production. In contrast to the hydrogen emissions, the CH(*A*^2^Δ) yield does not exhibit distinct isolated maxima but instead forms a broad composite structure with slowly varying intensity above approximately 30 eV. The broad underlying contribution may partly arise from dissociative decay following valence-shell photoionization, in addition to neutral-state dissociation.

The excitation function of the C_2_(*d*^3^Π_g_) emission exhibits a simpler structure dominated by broad maxima centered at approximately 25, 29, and 34 eV. Compared with the CH(*A*^2^Δ) channel, the C_2_(*d*^3^Π_g_) profile is more localized energetically and shows less contribution from low-energy excitation channels. The appearance energy estimated for the C_2_(*d*^3^Π_g_) emission is found to be 20.2 ± 0.5 eV, similar to the threshold energies observed for the Balmer emissions. Together with the somewhat localized excitation profile, this suggests that the formation of electronically excited C_2_ fragments is associated with relatively energetic dissociation pathways involving fragmentation of the carbon skeleton of the molecule.

The different shapes of the CH(*A*^2^Δ) and C_2_(*d*^3^Π_g_) excitation functions indicate that these fragments are associated with different regions of the excited-state manifold accessed following VUV excitation of furan. Although both excitation functions are relatively broad and exhibit maxima near 34 eV, their different shapes indicate that the CH(*A*^2^Δ) and C_2_(*d*^3^Π_g_) fragments are associated with different subsets of the highly excited states populated following VUV excitation of furan.

### 2.3. Relative Fragment Emission Yields

**Figure 5:**
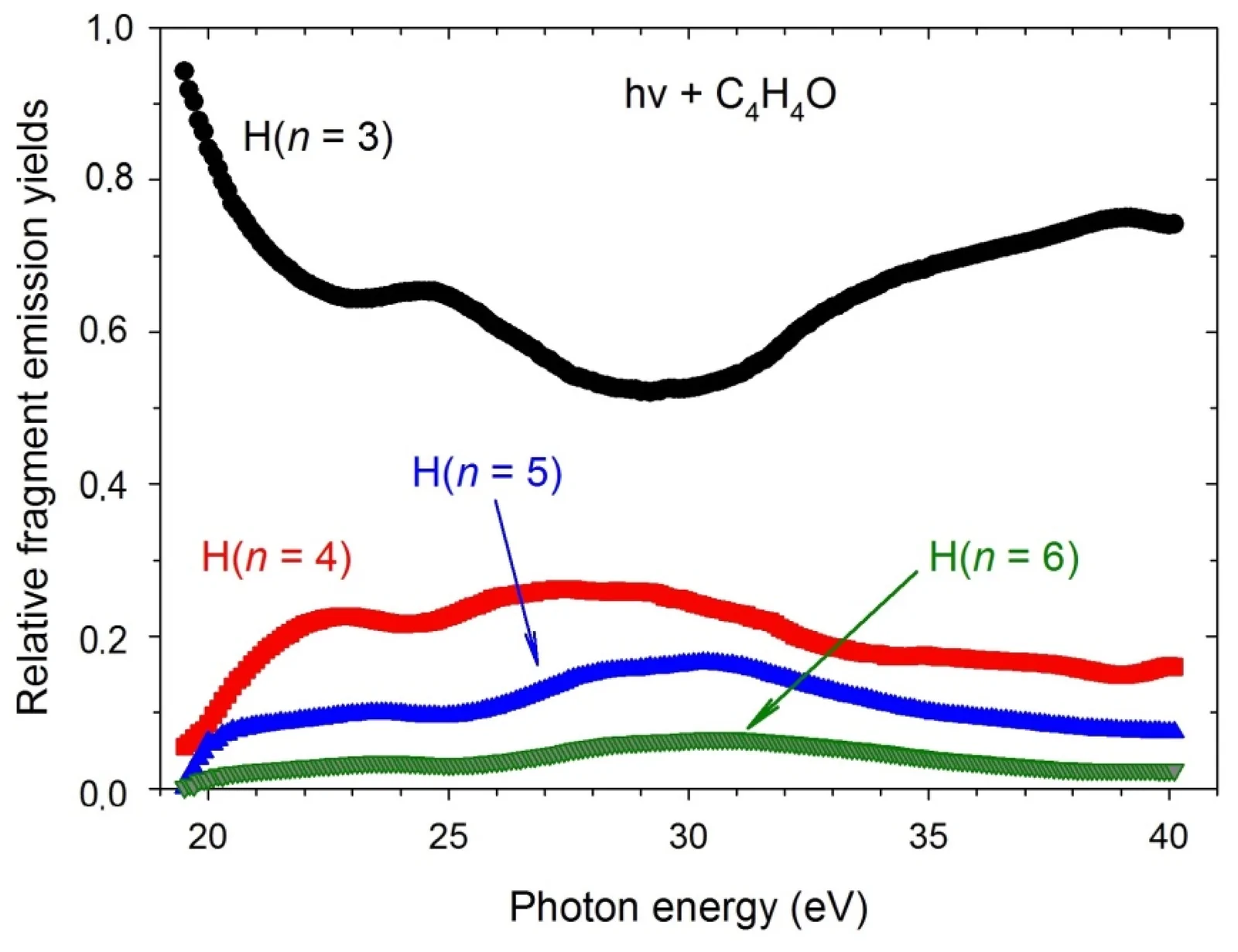
Relative fragment emission yields of the H(*n* = 3–6) emission channels to the total observed Balmer emission intensity as a function of photon energy in the VUV-induced dissociation of furan. The ratios were obtained by normalizing the smoothed emission yield curves of the individual Balmer lines to the summed Balmer emission intensity at each excitation energy.

**Figure 6:**
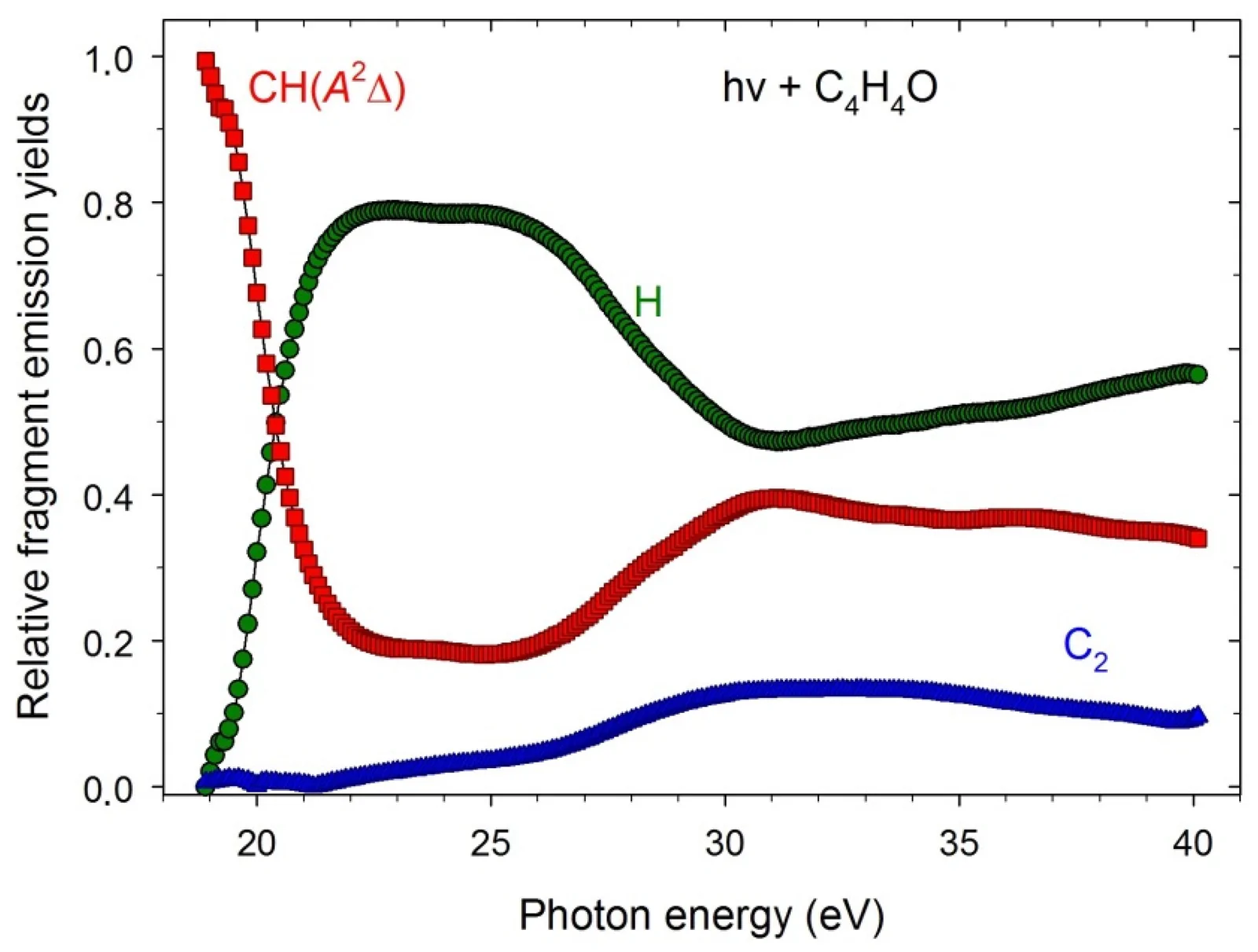
Relative fragment emission yields of the H, C_2_, and CH emission channels to the total observed emission intensity as a function of photon energy in the VUV-induced dissociation of furan.

Inspired by recent fragment-selective analyses in photodissociation spectroscopy [47], the photon-energy dependences of the H, C_2_, and CH fluorescence channels are compared here to probe the underlying excited-state dynamics. The relative fragment emission yields were defined as the ratio of the emission intensity of a given fragmentation channel to the summed intensities of all observed emissive fragmentation channels [47]. Although the present work does not provide a complete mapping of all emitting dissociation products, the observed fragment specific relative emission yield profiles reveal clear differences between direct fragmentation pathways and channels associated with more extensive fragmentation and potentially greater structural reorganization prior to dissociation. The comparison of atomic and molecular emission channels therefore provides complementary information on the decay of highly excited states populated in furan. These relative fragment emission yield curves are shown in Figure 5 and Figure 6. They were calculated from smoothed emission-yield curves to reduce statistical fluctuations and better illustrate the overall energy dependence of the competing fragmentation channels. Smoothing was performed using the Negative Exponential method, which applies Gaussian weights to neighboring data points. The Nearest Neighbors option was used with a sampling proportion of 0.10 and a third-degree polynomial. The obtained relative fragment emission yields do not represent absolute dissociation branching fractions, but rather relative contributions of the observed emission fragmentation channels, and are therefore interpreted here primarily as qualitative indicators of competition between the observed emission pathways. Nevertheless, their excitation-energy dependences provide useful insight into the fragmentation dynamics following VUV excitation.

The relative fragment emission yields of the individual Balmer emission channels were analyzed as a function of excitation energy to examine how the electronic-state distribution of the observed Balmer-line emission among the detected channels changes with excitation energy. Since the different Balmer lines originate from hydrogen atoms formed in different excited states, comparison of their relative emission intensities provides additional information on the distribution of excitation energy among the hydrogen fragments formed during dissociation. However, radiative cascades and state-dependent transition probabilities may contribute to the observed Balmer-line intensities, and the relative line yields are not interpreted as direct initial-state populations of the H(*n*) fragments.

The relative contributions of the observed individual Balmer emission channels to the total observed hydrogen emission are shown in Figure 5. The Hα line remains the dominant contribution throughout the investigated energy range. However, its relative intensity generally decreases between approximately 20 and 30 eV, with a small bump in the 23–27 eV region, while the higher-order Balmer lines become more prominent. Since the production of H(*n* = 4–6) requires progressively larger excitation energies of the hydrogen fragment, this behavior indicates increasing population of dissociation pathways capable of producing more highly excited hydrogen fragments. In particular, the Hγ and Hδ channels become noticeably enhanced in the 25–32 eV region, indicating changes in the dissociation dynamics and in the partitioning of excitation energy among the hydrogen fragments. In the 27–31 eV range, the relative contributions of the individual Balmer channels remain nearly constant despite variations in the absolute emission yields, suggesting that the relative population of the emitting hydrogen states changes only weakly in this energy region. The pronounced excitation energy dependence of the relative Balmer contributions suggests that changes in fragmentation dynamics contribute to the observed behavior.

Above approximately 32–33 eV, the relative contribution of Hα increases again, while those of Hβ, Hγ, and Hδ gradually decrease. This behavior may reflect the gradual opening of an additional dissociation channel, possibly associated with the population of highly excited ionic states and their accompanying correlation satellite states, which become increasingly accessible in this energy region, as discussed in Section 2.5. Such a mechanism may preferentially populate lower excited hydrogen states, although possible contributions to the Lyman series cannot be assessed within the spectral range of the present measurements.

The relative H, CH(*A*^2^Δ), and C_2_(*d*^3^Π_g_) emission yields are compared in Figure 6. The CH emission exhibits a dominant contribution close to its appearance threshold, but its relative contribution decreases rapidly between approximately 20 and 23 eV, where the hydrogen-emission channel becomes dominant. The hydrogen emission then exhibits its largest relative contribution in the 22–27 eV region before decreasing again as the relative contributions of the molecular fragment channels increase. Above approximately 30 eV, the hydrogen emission contribution increases moderately once more, which appears to correlate primarily with the increasing relative contribution of the H(*n* = 3) channel observed in Figure 5. In contrast, the C_2_(*d*^3^Π_g_) channel becomes increasingly important only at higher excitation energies, suggesting that its formation is associated with higher energy dissociation pathways. As discussed in Section 2.5, the increasing contribution of highly excited ionic states may provide a possible explanation for this behavior. The CH(*A*^2^Δ) emission yield contains both lower- and higher-energy structures, whereas the C_2_(*d*^3^Π_g_) yield is dominated by broader higher-energy contributions.

It is noteworthy that the relative Balmer emission yields shown in Figure 5 differ substantially from the relative H/CH/C_2_ emission yields presented in Figure 6. The Balmer yields mainly reflect the distribution of excitation energy among different electronic states of the hydrogen fragment, whereas the H/CH/C_2_ relative emission yields probe competition between fundamentally different fragmentation pathways of the parent molecule. The two sets of relative emission yields therefore provide complementary information on the dissociation dynamics following VUV excitation of furan.

### 2.4. Appearance Energies and Fragmentation Channels

**Figure 7:**
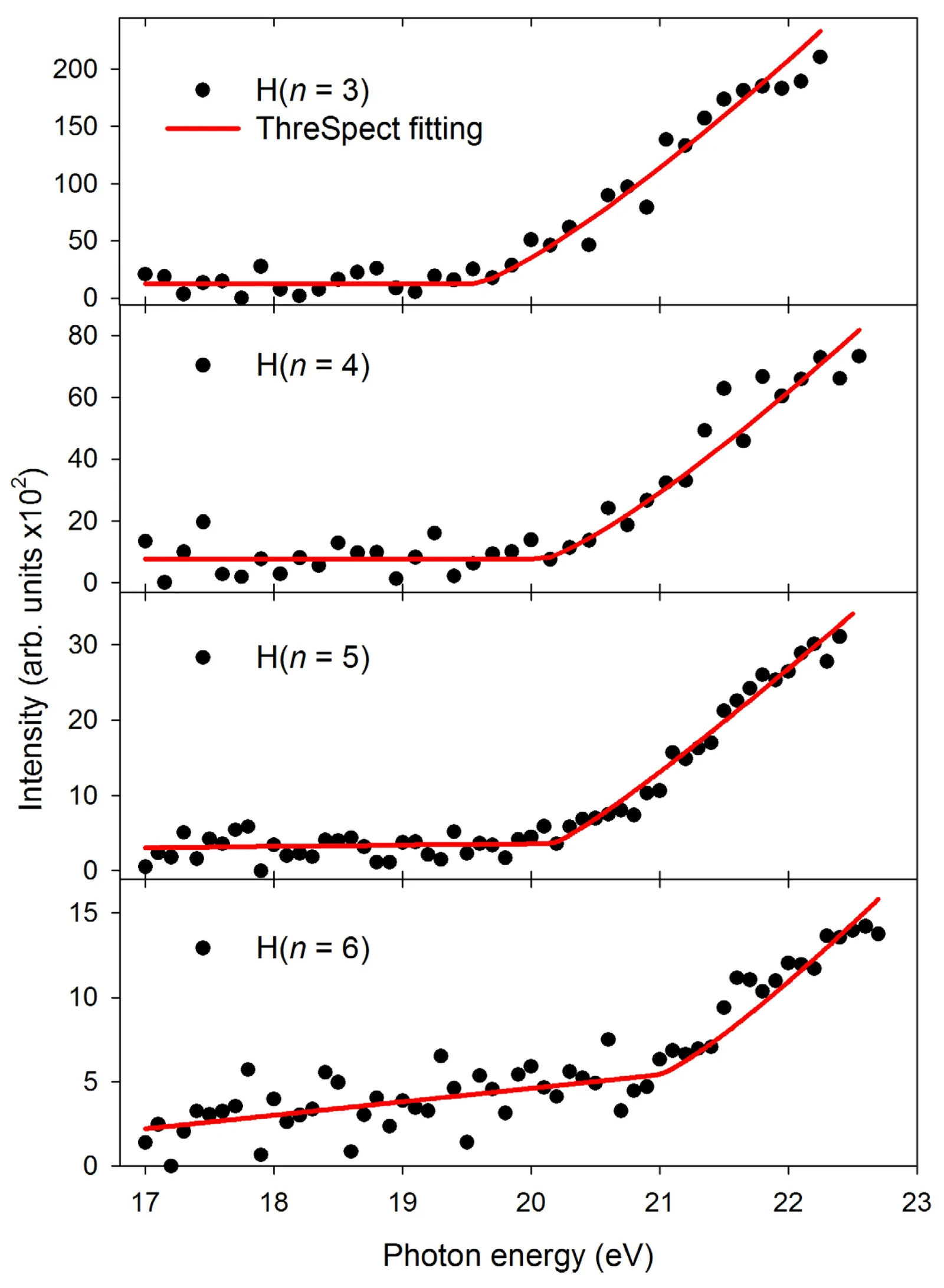
Near-threshold emission-yield curves of H(*n* = 3–6) fragments formed following VUV excitation of furan. Black dots represent the experimental data, and red lines show the ThreSpect fits.

The appearance energies (AE) were determined by fitting the near-threshold regions of the experimental emission-yield curves using the ThreSpect program [48]. The appearance energy, a scaling factor, and a threshold exponent were treated as free fitting parameters. The experimental data and the corresponding fits are shown in Figure 7. Additional uncertainties related to energy calibration and instrumental stability are estimated to be approximately ±0.1 eV. This fitting procedure has previously been applied successfully in electron- and photon-induced fragmentation studies [49,50].

The Balmer series emissions observed in the present experiment indicate the formation of electronically excited hydrogen atoms during the dissociation of furan. In particular, the Hα emission corresponds to the *n* = 3→ 2 radiative transition of atomic hydrogen and therefore requires formation of H(*n* = 3) fragments with an excitation energy of 12.09 eV [44]. The corresponding dissociation energy limit estimated for the production of H(*n* = 3) is 17.29 eV (Table 1), while the experimental appearance (threshold) energy determined from the threshold fit is 19.6 ± 0.3 eV. The appearance energies of approximately 20.1 to 21.0 eV were obtained for the higher excited hydrogen fragments H(*n* = 4–6), which are also clearly larger than the estimated dissociation energy limits given in Table 1.

The appearance energies observed for the Balmer emissions are systematically higher than the corresponding dissociation energy limits. This difference indicates that production of excited hydrogen atoms does not proceed through simple cleavage of a single C–H bond. Previous thermochemical studies have shown that direct cleavage of the C–H bond at the α-carbon atom in furan is energetically unfavorable due to its relatively high bond dissociation energy of about 120 kcal/mol (5.2 eV) [31]. The observed excess energy suggests that formation of H(*n* = 3–6) fragments is unlikely to proceed via simple bond cleavage and is more plausibly associated with multistep dissociation pathways involving ring opening, hydrogen migration, and decomposition of highly excited intermediate structures prior to hydrogen emission. Similar mechanisms were proposed previously in theoretical and collision-induced fragmentation studies of furan, where hydrogen rearrangement and carbene-like intermediates were found to play an important role in the dissociation dynamics [40,41]. While the present measurements do not identify the intermediate structures involved, the observed appearance energies and excitation-energy dependences are consistent with such multistep dissociation scenarios. The excitation functions of all Balmer lines exhibit two characteristic maxima located near 22.0 and 25.0 eV (Table 1). The presence of these structures indicates that hydrogen formation proceeds through several overlapping excitation channels associated with different regions of the highly excited-state manifold. As discussed in Section 2.5, the relative importance of neutral superexcited states and highly excited ionic states may vary across this energy range. Since production of H(*n* = 3–6) requires a substantial fraction of the available energy to be transferred into electronic excitation of the hydrogen atom, the Balmer emissions probe more energetic dissociation channels than the CH(*A*^2^Δ) emission, whose excitation energies are significantly lower.

The CH(*A*^2^Δ) and C_2_(*d*^3^Π_g_) emissions provide complementary information on the dissociation pathways accessed following VUV excitation of furan. In contrast to the Balmer emissions, the CH(*A*^2^Δ) channel exhibits a substantial contribution already at excitation energies below 20 eV and remains observable throughout the investigated energy range. The broad excitation profile extending from approximately 17 to 40 eV indicates that electronically excited CH radicals are produced through several competing dissociation pathways associated with different regions of the highly excited-state manifold.

Previous studies of electron-induced dissociation as well as theoretical investigations of neutral furan fragmentation have suggested that decomposition proceeds through initial C–O bond cleavage followed by ring opening and formation of highly excited open-chain intermediates [28,40] Subsequent decomposition of these intermediates can lead to CH production through several competing fragmentation channels involving the loss of H, H_2_, O, CO, or larger neutral fragments [28,40]. Although the present measurements do not identify the intermediate species involved, the broad photon-energy distribution of the CH(*A*^2^Δ) emission is consistent with such multistep dissociation processes. However, it may also reflect contributions from a dense manifold of overlapping highly excited ionic states, in particular from correlation satellite states. At the same time, formation of an electronically excited CH fragment requires substantial fragmentation and reorganization of the carbon skeleton. The relatively low appearance energy suggests that formation of electronically excited CH fragments becomes accessible already in the lowest energy region of the excited-state manifold populated following VUV excitation, where neutral superexcited states are expected to provide the dominant contribution.

The C_2_(*d*^3^Π_g_) emission exhibits a markedly different excitation-energy dependence. Its appearance energy is close to 20 eV and its excitation profile is dominated by broad structures centered in the 25–39 eV region. In contrast to the CH(*A* ^2^Δ) channel, the relative contribution of the C_2_(*d*^3^Π_g_) emission becomes increasingly important only at higher excitation energies. These observations suggest that formation of electronically excited C_2_ fragments may be associated with relatively energetic dissociation pathways and may become increasingly favorable as larger amounts of internal energy are deposited into the molecule. Although the present data do not permit identification of specific reaction mechanisms, the increasing importance of the C_2_(*d*^3^Π_g_) channel at higher excitation energies is consistent with theoretical predictions that fragmentation of the molecular skeleton of furan becomes increasingly important following the initial ring-opening processes [40].

The distinct photon energy dependences of the CH(*A*^2^Δ) and C_2_(*d*^3^Π_g_) channels indicate that these fragments are associated with different regions of the highly excited-state manifold accessed following VUV excitation of furan.

### 2.5. Neutral and Ionic Contributions to Fragment Fluorescence

The appearance energies determined for the H(*n*) fragments exceed the corresponding minimum dissociation-energy limits listed in Table 1. Such differences suggest that the observed fragmentation cannot be described solely in terms of direct C–H bond cleavage and that additional molecular rearrangement precedes dissociation. Similar conclusions were reached in the previous PIFS studies of heterocyclic molecules [46,51,52]. In particular, studies of isoxazole demonstrated that assessment of competing neutral and ionic dissociation pathways requires explicit consideration of their energetic thresholds and that autoionization-mediated dissociation may be excluded only when its onset lies significantly above the experimentally observed excitation range [46,51]. In pyridine, H(*n* = 4–6) fragments were observed at 19.1–19.6 eV, while the corresponding dissociation limits were estimated at 17.34–18.07 eV. Quantum-chemical calculations showed that hydrogen migration and ring-opening processes play a key role in the formation of electronically excited fragments [50]. Although analogous calculations are not available for furan, the excess energies observed here are consistent with the occurrence of molecular rearrangement prior to fragmentation.

The interpretation of these rearrangement processes may be viewed in the broader context of molecular superexcitation. Hatano emphasized that superexcited states constitute neutral excited states located above the first ionization threshold and may decay through competing dissociation and autoionization channels [43]. For many molecular systems, such states form Rydberg series converging to ionic state limits, establishing a close connection between neutral and ionic excitation pathways [43]. In the case of isoxazole, it was further noted that the dissociation dynamics of Rydberg-type superexcited states are largely governed by the corresponding ionic core configurations [51]. Because the Rydberg electron is weakly bound and spatially diffuse, structural rearrangements leading to ring opening and hydrogen migration are primarily determined by the potential energy surface of the ionic core rather than by the excited electron itself [51]. Although no analogous calculations are presently available for furan, this picture provides a plausible framework for interpreting the rearrangement processes inferred from the appearance energies reported in Table 1.

An additional aspect that should be considered concerns the electronic character of the states populated in the 17–48 eV photon energy range. Photoabsorption and photoelectron studies of furan revealed a dense manifold of ionic states extending over a broad energy range above the first ionization threshold (8.89 eV) [39,53]. In particular, synchrotron radiation photoelectron spectroscopy resolved additional correlation (satellite) states accompanying the principal valence ionization bands, demonstrating that many-electron excitations constitute an intrinsic part of the valence ionization spectrum of furan [53,54]. Subsequent SAC-CI calculations by Ehara et al. [55] showed that the simple one-hole (1h) description progressively breaks down in the inner-valence region because the principal ionization channels interact strongly with shake-up configurations. In particular, the intensities associated with the 3*b*_2_ and 5*a*_1_ ionizations are redistributed over numerous satellite states, reflecting substantial two-hole–one-particle (2h–1p) character [55]. Similar electron-correlation effects have been extensively documented for benzene and other aromatic molecules [56,57,58,59,60]. In benzene, a prominent correlation satellite was identified near 16.7 eV ionization energy [56], while Green’s-function and ADC calculations demonstrated that the importance of 2h–1p configurations increases rapidly in the inner-valence region and that the simple one-hole description progressively breaks down above approximately 18 eV [57,58,59,60].

These considerations may be relevant to the higher-energy part of the excitation functions shown in Figure 3 and Figure 4. In pyridine, the minimum energy required for production of H(*n* = 4) following autoionization and subsequent dissociation of the molecular cation was estimated to be 26.9 eV [52]. An analogous estimate can be performed for furan. Electron-impact mass-spectrometric measurements yielded an appearance energy of 12.80 ± 0.10 eV [29] for the ionic dissociation channel producing C_4_H_3_O^+^ + H. Assuming formation of the same ionic co-fragment together with an electronically excited hydrogen atom, addition of the H(*n* = 3) excitation energy of 12.09 eV [44] gives an estimated energetic threshold of 24.89 eV for the C_4_H_3_O^+^ + H(*n* = 3) + e^−^ channel. The corresponding estimates for H(*n* = 4–6) are 25.55, 25.86, and 26.02 eV, respectively. These values fall within the photon-energy region in which the second broad structures in the Balmer excitation functions and pronounced changes in the relative emission channel contributions are observed (Figure 5 and Figure 6). Although this correlation does not constitute evidence for an ionic dissociation pathway, it indicates that such channels become energetically accessible in the same energy range. Neutral doubly excited states located below the double-ionization threshold may also contribute to the formation of electronically excited neutral fragments through dissociative decay. The double-ionization threshold of furan (25.18 eV) [61] lies within the investigated photon-energy range. Above this threshold, fragmentation of highly excited dicationic states may represent an additional source of electronically excited neutral fragments, although the present measurements do not allow this possibility to be evaluated separately. The relative importance of the underlying excitation mechanisms is expected to depend on photon energy. Below the estimated thresholds of 24.89–26.02 eV, the corresponding C_4_H_3_O^+^ + H(*n*) + e^−^ fragmentation channels are energetically inaccessible, and dissociation of neutral superexcited states may contribute to the observed H(*n*) emission. Above approximately 26 eV, all these ionic fragmentation channels become energetically accessible. At higher photon energies, direct valence-shell photoionization may populate not only the principal ionic states but also correlation satellite states with substantial two-hole–one-particle (2h–1p) character, which are known to accompany the principal valence ionization spectrum of furan [54,55]. Dissociative decay of these highly excited ionic states may therefore contribute to the observed fragment fluorescence. However, since the PIFS technique detects only fluorescence from the final neutral fragments and provides no coincident information on photoelectrons or ionic products, the relative contributions of neutral superexcited states, principal ionic states, and correlation satellite states cannot be determined from the present measurements.

## 3. Materials and Methods

**Figure 8:**
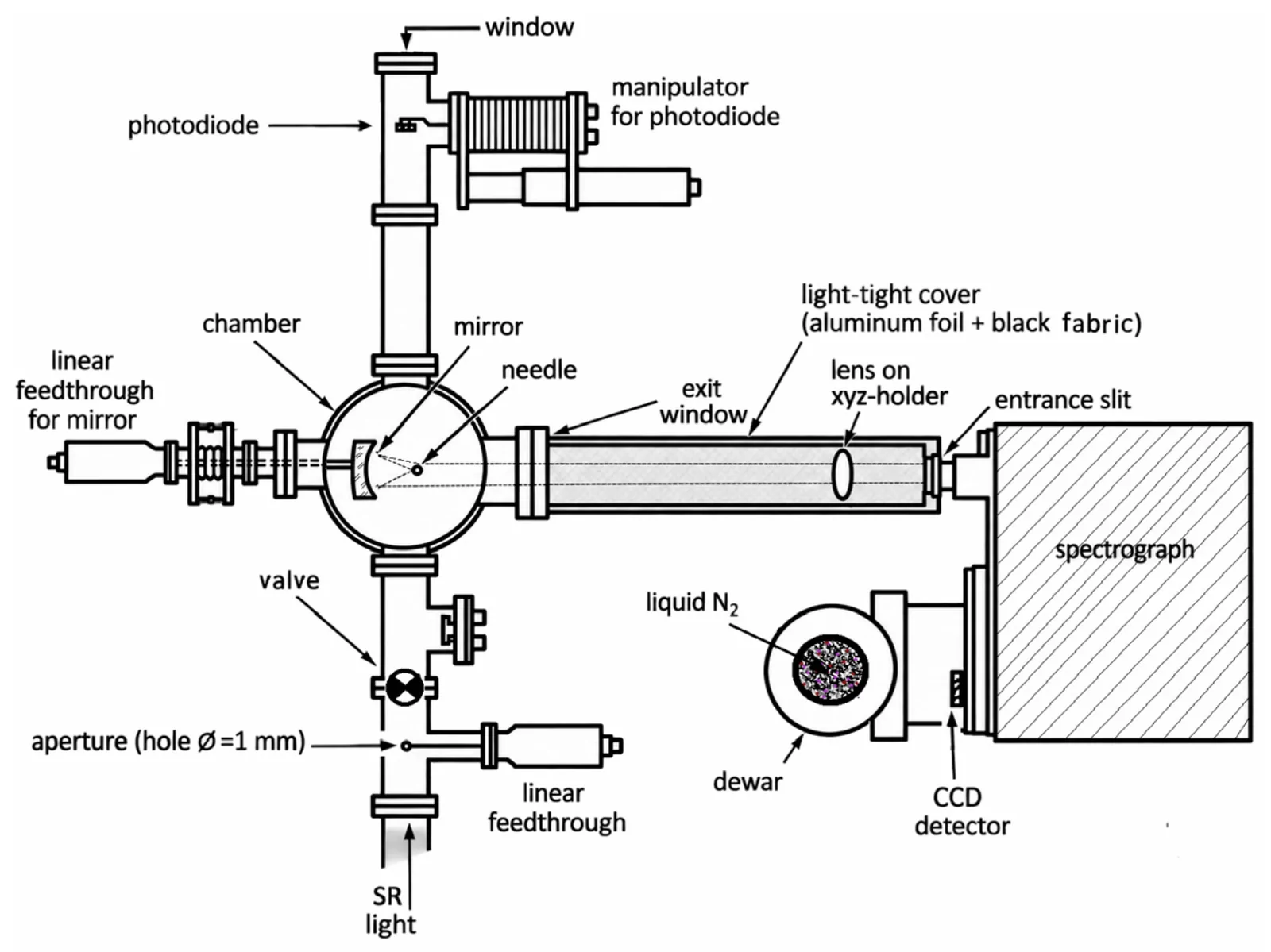
Schematic of the experimental setup used for photon-induced fluorescence measurements of furan. Adapted from Ref. [45].

The photon-induced fluorescence spectroscopy (PIFS) [45,62] measurements of furan were carried out at the Gas Phase Photoemission beamline [63] at the Elettra Synchrotron Radiation Facility in Trieste, Italy. The combination of this technique with synchrotron-radiation excitation has been successfully applied by us before in studies of other five- and six-membered heterocyclic molecules [45,46,51,52]. A schematic of the experimental setup used in the present experiment is shown in Figure 8. A detailed description of this apparatus is presented elsewhere [45,62] and only a concise summary is given here including data specifically related to furan.

Briefly, the beamline consisted of an undulator source and a spherical-grating monochromator equipped with a movable planar premirror and two refocusing mirrors. It delivered intense, well-collimated radiation in the photon-energy range from 13 eV to above 900 eV. Five gratings were available to cover different photon-energy ranges. In the present experiment, the low-energy gratings, G1 and G6, were used. The G1 and G6 gratings covered photon-energy ranges of 13–70 eV and 13–25 eV, respectively, G1 being used for higher resolving power, G6 for higher flux and better higher order rejection at lower photon energies. The photon-energy scale was calibrated before the measurements using selected narrow autoionizing resonances of noble gases (He, Ar, Ne) and N_2_ [45]. The photon-energy bandwidth under the experimental conditions used in the present measurements was approximately 20 meV (FWHM), as determined from the widths of the noble-gas resonances. The uncertainty of the absolute photon-energy scale was less than 20 meV. The monochromatized synchrotron beam was directed to the experimental station, where it intersected an effusive molecular jet coming from a hypodermic needle. The photon flux was monitored simultaneously during data acquisition using a calibrated photodiode.

The furan sample (Sigma-Aldrich, St. Louis, MO, USA; purity > 99%) was purified by repeated freeze–pump–thaw cycles until no further gas release was observed during melting. At room temperature, the vapor pressure of furan (493 mmHg at 20 °C) [64] was sufficient to provide a stable gas flow without heating of the sample. The base pressure of the vacuum system, without gas introduction, was less than 2 × 10^−7^ mbar. During the excitation-function measurements, a chamber pressure of approximately 5.9 × 10^−5^ mbar was recorded, whereas during acquisition of the fluorescence spectra at 62 eV, the pressure was typically (1.2–1.3) × 10^−4^ mbar. The pressure in the interaction region was estimated to be about 30 times higher.

The fluorescence emitted from the interaction region was collected by a spherical mirror and focused by a lens onto the entrance slit of a 0.5 m Minuteman 305MV spectrograph (Minuteman Laboratories Inc., Acton, MA, USA). The light was dispersed by a 1200 lines/mm grating and detected with a liquid-nitrogen-cooled CCD camera (Spec-10:100B, Princeton Instruments, Trenton, NJ, USA), allowing the simultaneous observation of a 40 nm wide spectral window.

Two different measurement modes were used. In the first mode, high-resolution fluorescence emission spectra were recorded at fixed incident photon energy of 62 eV to identify the emitting fragments. This energy was selected because all dissociation channels discussed here were energetically accessible, fragment emission was relatively intense, and second-order radiation did not affect the measurements. It also allows comparison with our previous emission spectra of tetrahydrofuran and isoxazole recorded at the same photon energy [45,46]. The complete spectrum over a wide wavelength range was obtained by combining a series of consecutive 40 nm scans. Individual CCD exposures lasted 300 s. For several wavelength windows, five consecutive exposures were recorded, corresponding to a total integration time of 1500 s per window. Fewer exposures were used in other spectral regions. All known sources of background, as described in Refs. [45,47] were carefully identified and subtracted from the recorded spectra. After background subtraction, all spectra were corrected for the wavelength-dependent response of the optical detection system [45]. As a result, a complete spectrum in the 300–677 nm range was obtained with an optical resolution Δλ = 0.5 nm (FWHM). The wavelength scale was calibrated using the positions of the observed Hα to Hε lines of the Balmer series with accuracy better than 0.1 nm.

In the second mode, the photon-energy dependence of the emission yield (excitation function) of selected transitions was measured. The spectrograph was set to record a 40 nm wavelength window, and fluorescence spectra were recorded while scanning the incident photon energy over ranges extending from 17 to 47 eV, with energy steps of 0.10, 0.15, or 0.50 eV, depending on the measurement. For the hydrogen emission yields, Hα (656.3 nm) and Hβ (486.1 nm) were measured in separate windows over 17–40 eV, using an energy step of 0.15 eV and an acquisition time of 10 s per photon energy. Hγ (434.0 nm) and Hδ (410.2 nm) were recorded simultaneously in the same window centred at 422 nm, over the photon-energy range of 17–47 eV, with an energy step of 0.10 eV and an acquisition time of 70 s per photon energy. For the other emission channels, acquisition times typically ranged from 30 to 600 s per photon energy. Each spectrum was corrected for the wavelength-dependent sensitivity of the optical detection channel [45]. All identified sources of background were carefully subtracted from each recorded spectrum. The intensities of atomic lines and molecular bands were obtained by integrating the counts over their full spectral profiles. The resulting emission yields were normalized to the incident photon flux and corrected for the wavelength-dependent sensitivity of the optical detection channel [45].

## 4. Conclusions

The dissociation dynamics of furan following VUV excitation have been investigated using photon-induced fluorescence spectroscopy. Dispersed emission spectra revealed electronically excited hydrogen atoms together with the CH(*A*^2^Δ, *B*^2^Σ^−^, *C*^2^Σ^+^) and C_2_(*d*^3^Π_g_) fragments as the dominant emissive photodissociation products. The excitation functions measured between 17 and 47 eV showed broad and structured energy dependences, reflecting the participation of overlapping manifolds of highly excited neutral and ionic states in the fragmentation dynamics.

Threshold analyses demonstrated that the appearance energies of the Balmer emissions are systematically higher than the corresponding dissociation energy limits. The excess of the measured appearance energies over the minimum thermochemical limits is consistent with, but does not uniquely establish, multistep dissociation involving structural rearrangement. The photon energy dependences of the H(*n* = 3–6), CH(*A*^2^Δ), and C_2_(d^3^Π_g_) channels are consistent with previous theoretical and experimental studies suggesting the importance of ring opening and hydrogen migration processes during furan fragmentation.

Analysis of the relative contributions of the observed emission channels revealed substantial differences between the hydrogen, CH, and C_2_ channels. The CH(*A*^2^Δ) emission contributes significantly already at the lowest photon energies accessible in the present experiment and remains important throughout the investigated energy range, whereas the C_2_(*d*^3^Π_g_) channel becomes increasingly important only at higher excitation energies. The Balmer emission channels exhibit pronounced variations in their relative contributions, indicating changes in the partitioning of excitation energy among the hydrogen fragments. All these observations suggest that different regions of the excited-state manifold preferentially populate different dissociation pathways and fragment excitation channels. The relative increase in the H(*n* = 3) contribution above 30 eV may reflect an increasing contribution from fragmentation following population of highly excited ionic states, including correlation satellite states.

The present results extend previous studies of furan fragmentation by providing direct information on the formation of electronically excited neutral fragments following VUV excitation. More generally, the results demonstrate that fragment selective optical emission spectroscopy, combined with excitation-energy-dependent measurements, provides a useful approach for probing the dissociation dynamics of highly excited states leading to the formation of excited neutral fragments in heterocyclic molecules.

## Figures and Tables

**Table 1:** Appearance energies, characteristic energy maxima of the Gaussian components observed in the hydrogen excitation function curves (Figure 3), dissociation energy limits, and excitation energies of the H(*n*), *n* = 3–6 fragments (all in eV). The quoted uncertainties were estimated from the fitting procedure.

Fragment	Appearance Energy	Energy of the 1st Band	Energy of the 2nd Band	Dissociation Energy Limit	Excitation Energy [44]
H(*n* = 3)	19.6 ± 0.3	21.8 ± 0.3	24.5 ± 0.2	17.29	12.09
H(*n* = 4)	20.1 ± 0.5	22.3 ± 0.4	25.2 ± 0.6	17.95	12.75
H(*n* = 5)	20.2 ± 0.6	22.4 ± 0.4	25.2 ± 0.4	18.26	13.06
H(*n* = 6)	21.0 ± 1.0	22.5 ± 0.5	25.3 ± 0.7	18.42	13.22

**Table 2:** Characteristic energy regions obtained from Gaussian decomposition of the excitation function curves for the H(*n*), CH(*A*^2^Δ), and C_2_(*d*^3^Π_g_) fragmentation channels in furan. The quoted energies are based on the maxima of the fitted Gaussian components. For H(*n*), the values of 28.0 and 33.1 eV are the mean positions of the corresponding components fitted to the four Balmer emission-yield curves. Uncertainties were estimated from the fitting procedure.

H(*n*)	CH(*A*^2^Δ)	C_2_(*d*^3^Π_g_)
	17.3 ± 0.3	
	19.4 ± 0.3	
21.8–22.5 *	22.2 ± 0.5	22.5 ± 0.5
24.5–25.3 *	24.2 ± 0.4	24.5 ± 0.5
28.0 ± 0.9	27.9 ± 0.9	28.5 ± 0.6
33.1 ± 0.9	32.7 ± 0.9	33.6 ± 0.7

* The individual peak maxima are listed in Table 1.

## Data Availability

The data supporting the findings of this study are available from the corresponding author upon reasonable request.

## Abbreviations

The following abbreviations are used in this manuscript:

CCD	charge-coupled device
FWHM	full width at half maximum
PIFS	photon-induced fluorescence spectroscopy
VUV	vacuum ultraviolet
